# Enhanced renal clearance and impact on vancomycin pharmacokinetic parameters in patients with hemorrhagic stroke

**DOI:** 10.1186/s40560-019-0408-y

**Published:** 2019-11-21

**Authors:** Kathryn A. Morbitzer, Denise H. Rhoney, Kelly A. Dehne, J. Dedrick Jordan

**Affiliations:** 1 Division of Practice Advancement and Clinical Education, UNC Eshelman School of Pharmacy, University of North Carolina at Chapel Hill, Chapel Hill, NC USA; 2 Department of Pharmacy, UNC Health Care, Chapel Hill, NC USA; 3 Division of Neurocritical Care, Departments of Neurology and Neurosurgery, University of North Carolina School of Medicine, 170 Manning Drive, Physician Office Building 2118, Chapel Hill, NC 27599 USA

**Keywords:** Subarachnoid hemorrhage, Intracerebral hemorrhage, Augmented renal clearance, Creatinine clearance, Vancomycin

## Abstract

**Background:**

The majority of patients with hemorrhagic stroke experience enhanced renal clearance or augmented renal clearance (ARC). The purpose of this study was to determine the impact of enhanced renal clearance or ARC on vancomycin pharmacokinetic (PK) parameters.

**Methods:**

This was a post hoc analysis of a prospective study of adult patients with aneurysmal subarachnoid hemorrhage (aSAH) or intracerebral hemorrhage (ICH) admitted to the neurosciences intensive care unit who received vancomycin. Creatinine clearance (CrCl) was measured and also estimated using the Cockcroft-Gault equation. Predicted PK parameters were compared with calculated PK parameters using serum peak and trough concentrations.

**Results:**

Seventeen hemorrhagic stroke patients met inclusion criteria. All patients experienced enhanced renal clearance on the day that the vancomycin concentrations were obtained, and 12 patients (71%) experienced ARC. The mean calculated elimination rate constant was significantly higher than the predicted value (0.141 ± 0.02 vs. 0.087 ± 0.01 h^−1^; *p* = 0.004) and the mean calculated half-life was significantly lower than the predicted half-life (6.5 ± 0.9 vs. 8.7 ± 0.6 h; *p* = 0.03).

**Conclusions:**

Patients with hemorrhagic stroke and enhanced renal clearance displayed PK alterations favoring an increased elimination of vancomycin than expected. This may result in underexposure to vancomycin, leading to treatment failure.

## Introduction

Patients with hemorrhagic stroke have high disability and mortality rates [1, 2]. In addition to the direct effects of the initial bleeding event and secondary neurologic complications, patients with hemorrhagic stroke are predisposed to medical complications that can have a negative impact on patient outcomes and increase cost of care [3–5].

One of the most common medical complications that occur in patients with hemorrhagic stroke is infection [3, 6]. The development of infections in this patient population is of significant concern as infections have been found to be one of the strongest drivers of length of stay and readmission within 30 days [7–9]. Vancomycin is a primarily renally eliminated antibiotic that undergoes routine drug monitoring to optimize clinical efficacy and limit toxicity in this patient population.

It has been previously reported that the vast majority of patients with hemorrhagic stroke experience a hyperdynamic state resulting in enhanced renal clearance (defined as a measured CrCl greater than that calculated based on standard equations) and/or augmented renal clearance (ARC) (defined as a measured CrCl greater than or equal to 130 mL/min/1.73 m^2^) [10, 11] However, to our knowledge, no studies exist examining the impact of these changes in renal clearance on renally eliminated medication pharmacokinetic parameters in this patient population. This is an important concept as clinicians do not routinely adjust the dosing regimen of renally eliminated medications to account for enhanced renal clearance or ARC, which may result in subtherapeutic concentrations of these medications and undertreatment of infectious complications. Therefore, the purpose of this study was to determine whether patients experiencing enhanced renal clearance or augmented renal clearance with hemorrhagic stroke had alterations in vancomycin pharmacokinetic parameters when compared with those predicted based on population-based equations.

## Materials and methods

### Patients

This was a prospective, observational post hoc analysis study that included adult patients with ICH or aSAH admitted to UNC Hospitals Neurosciences Intensive Care Unit [10]. Ethical approval was obtained from the institutional review board. Patients had an expected ICU length of stay (LOS) greater than 48 h, received vancomycin, and had one vancomycin peak steady-state concentration and one vancomycin trough steady-state concentration obtained. Patients were excluded if an indwelling urinary catheter was not used as part of standard management, they had pre-existing renal dysfunction (CKD stages 3–5), they had an admission serum creatinine > 1.4 mg/dL, they had a history of nephrectomy or renal transplant, they had a BMI < 18 kg/m^2^, or if they were pregnant. Management of ICH and aSAH at UNC Hospitals was consistent with published guidelines [12, 13].

### Vancomycin dosing regimens

Clinical pharmacists are consulted for vancomycin initiation for patients admitted to the Neurosciences Intensive Care Unit at UNC Hospitals. The consulted clinical pharmacists are responsible for determining the vancomycin dosing regimen. The goal serum vancomycin trough concentration at steady-state is 10–20 ug/mL, depending on the indication. Clinical pharmacists are also responsible for determining the optimal time to obtain steady-state vancomycin serum concentrations, which is generally considered to be at least four times the half-life of the drug after administration.

### Interventions

Demographic and outcome data, including age, gender, admission diagnosis, admission height, admission weight, admission Glasgow Coma Scale (GCS) score, admission Sequential Organ Failure Assessment (SOFA) score, ICU (intensive care unit) and hospital LOS, and mortality were recorded prospectively. Specifically, for the aSAH patients, the Hunt and Hess scale grade, modified Fisher scale grade, and occurrence of symptomatic vasospasm were recorded. For the ICH patients, admission ICH score and ICH volume were collected. Serum creatinine, urine creatinine, and urine volume were recorded daily. Data collected for characterizing the vancomycin regimen include vancomycin dose, vancomycin frequency, vancomycin serum concentrations, and fluid balance.

An 8-h urine collection was the primary method of measuring renal function. CrCl was also calculated based on the Cockcroft-Gault equation. Urine was collected daily via the indwelling urinary catheter between 10:00 and 18:00, following which laboratory analysis determined the urinary volume and urinary creatinine concentration. Concurrent daily serum creatinine concentrations were obtained, following which CrCl was calculated using the following formula:

$$ \mathrm{CrCl}=\left[{\mathrm{U}}_{\mathrm{Cr}}\times {\mathrm{U}}_{\mathrm{vol}}\right]/\left[{\mathrm{S}}_{\mathrm{Cr}}\times {\mathrm{T}}_{\mathrm{min}}\right] $$

CrCl determined by the Cockcroft-Gault method was calculated using the following formula:

$$ \mathrm{CrCl}=\left[\left(140-\mathrm{Age}\right)\times \left(\mathrm{Weight}\ \left(\mathrm{kg}\right)\right)\times \left(0.85\ \mathrm{if}\ \mathrm{Female}\right)\right]/\left(72\times \mathrm{SCr}\ \left(\mathrm{mg}/\mathrm{dL}\right)\right) $$

CrCl and eGFR values were normalized to a body surface area of 1.73 m^2^, as per convention. Enhanced renal clearance was defined as a measured CrCl greater than the calculated CrCl via the Cockcroft-Gault equation. ARC was defined as an 8-h CrCl greater than or equal to 130 mL/min/1.73 m^2^, given the previously determined association with subtherapeutic antimicrobial concentrations when using standard doses [14].

### Pharmacokinetic measures

To determine alterations in pharmacokinetic parameters of vancomycin therapy following an aSAH or ICH, predicted pharmacokinetic parameters based on population data were compared with pharmacokinetic parameters calculated based on available steady-state vancomycin serum peak and trough concentrations. Predicted pharmacokinetic parameters were calculated using the following equations [15]:

$$ {V}_{\mathrm{d}}=0.7\ \mathrm{L}\times \mathrm{actual}\ \mathrm{body}\ \mathrm{weight}\ \left(\mathrm{kg}\right) $$

$$ {K}_{\mathrm{e}}=0.00083\times \mathrm{CrCl}+0.0044 $$

where *V*_d_ is the volume of distribution, *K*_e_ is the first-order elimination rate constant, and CrCl is the estimated creatinine clearance based on the Cockroft and Gault equation [16]. Ideal body weight was used in the Cockroft and Gault equation, except if the actual body weight was less than the ideal body weight, then the actual body weight was used, or if the actual body weight was greater than 125% of the ideal body weight, then the adjusted body weight was used [17, 18]. The predicted pharmacokinetic parameters calculated by the equations were then used to determine the estimated vancomycin serum trough concentration, which was compared with the measured vancomycin serum trough concentration.

The following equations were used to calculate patient pharmacokinetic parameters based on steady-state vancomycin serum peak and trough concentrations [19]:

$$ {K}_{\mathrm{e}}=\left[\ln\ \left(C\max /C\min \right)\right]/\varDelta\ t $$

$$ {V}_{\mathrm{d}}=\left[\left(\mathrm{Dose}/t'\right)\ \left(1-{\mathrm{e}}^{-\mathrm{ket}'}\right)\right]/\left\{\mathrm{ke}\left[C\max -\left(C\min \times {\mathrm{e}}^{-\mathrm{ket}\hbox{'}}\right)\right]\right\} $$

where *C*max is the steady-state vancomycin serum peak concentration, *C*min is the steady-state vancomycin serum trough concentration, Δ *t* is difference in time between *C*max and *C*min within the same dosing interval, and *t*’ is the infusion time.

### Statistical analysis

Descriptive statistics were used to portray patient characteristics and vancomycin regimen characteristics. Continuous and ordinal variables are represented as mean ± standard deviation or median (interquartile range), and categorical variables are represented as *n* (%). The pharmacokinetic parameters were compared using the paired *t* test. Statistical significance was defined as a *p* value < 0.05. All analyses were performed with Stata version 14.2 (StataCorp LP, College Station, TX).

## Results

The patients eligible for inclusion into the study have been previously described [10]. During the study period, 64 aSAH patients and 60 ICH patients were admitted to the Neurosciences Intensive Care Unit. Seventeen patients were included in the study and received vancomycin, and had at least one vancomycin peak steady-state concentration and one vancomycin trough steady-state concentration obtained.

### Patient characteristics

Table 1 displays the patient characteristics for those included within the study. The majority of patients were female (65%) with a mean age of 63.3 ± 13.3 years. The median admission GCS was 9 (6–12), and the median admission SOFA score was 4 (3–5). The majority of patients had an admission diagnosis of aSAH (71%). The mean admission serum creatinine was 0.7 ± 0.1 mg/dL.

**Table 1:** Patient characteristics

Variable	Hemorrhagic stroke (*n* = 17)
Age (years), mean ± SD	63.3 ± 13.3
Weight (kg), mean ± SD	82.3 ± 15.4
Female gender, *n* (%)	11 (65)
Admission GCS, median (IQR)	9 (6–12)
Admission SOFA score, median (IQR)	4 (3–5)
Subarachnoid hemorrhage, *n* (%)	12 (71)
Hunt and Hess scale grade, median (IQR)	3 (2–4)
Modified Fisher grade, median (IQR)	3 (3–4)
Admission ICH score, median (IQR)	3.5 (3–4)
Admission ICH volume, median (IQR)	95.9 (59.7–136.5)
Admission SCr (mg/dL), mean ± SD	0.7 ± 0.1
Selected co-morbidities, *n* (%)	
Hypertension	11 (65)
Diabetes	1 (6)
Congestive heart failure	2 (12)

*GCS*, Glasgow Coma Scale; *SOFA*, Sequential Organ Failure Assessment; *ICH*, intracerebral hemorrhage; *SCr*, serum creatinine

### Vancomycin characteristics

Patient vancomycin characteristics are displayed in Table 2. The mean vancomycin dosing regimen was 15.1 ± 4.2 mg/kg every 8 (8–12) h. This provided a mean vancomycin serum trough concentration of 12.0 ± 3.6 ug/mL. The median daily fluid balance of 48 h prior to the vancomycin serum trough concentration was + 588.4 (− 52.2–1440.7) mL/day. The most common infection source for administration of vancomycin was pneumonia (53%).

**Table 2:** Vancomycin characteristics

Variable	Hemorrhagic stroke (*n* = 17)
Days between admission date and trough concentration, mean ± SD	7.4 ± 3.0
Dose (mg/kg), mean ± SD	15.1 ± 4.2
Frequency (h), median (IQR)	8 (8–12)
Serum trough concentration (ug/mL), mean ± SD	12 ± 3.6
Daily fluid balance of 48 h prior to serum trough concentration (mL/day), median (IQR)	+ 588.4 (− 52.2–1440.7)
Infection source, *n* (%)	
Pneumonia	9 (53%)
Empiric	4 (23.5%)
Tracheobronchitis	3 (17.6%)
Sepsis	1 (5.9%)

### Vancomycin pharmacokinetic parameters

Table 3 displays the predicted pharmacokinetic parameters based on population data and the pharmacokinetic parameters calculated from steady-state vancomycin serum peak and trough concentrations. The mean creatinine clearance calculated from urine creatinine measurements on the day that the vancomycin concentrations were obtained was significantly higher than the creatinine clearance calculated from the Cockcroft-Gault equation (116.7 ± 10.3 vs. 161.6 ± 16.7 mL/min; *p* = 0.001). All patients experienced enhanced renal clearance on the day that the vancomycin concentrations were obtained, and 12 patients (71%) experienced ARC. The mean calculated elimination rate constant was also significantly higher than the predicted value (0.141 ± 0.02 vs. 0.087 ± 0.01 h^−1^; *p* = 0.004), and the mean calculated half-life was significantly lower than the predicted half-life (6.5 ± 0.9 vs. 8.7 ± 0.6 h; *p* = 0.03). No difference was seen between the mean calculated volume of distribution and the mean predicted volume of distribution (71.8 ± 11.3 vs. 57.6 ± 2.6 L; *p* = 0.23).

**Table 3:** Pharmacokinetic parameters

Variable	Predicted value (*n* = 17)	Calculated value (*n* = 17)	*p* value
Creatinine clearance (mL/min), mean ± SD	116.7 ± 10.3	161.6 ± 16.7	0.001
Elimination rate constant (h^−1^), mean ± SD	0.087 ± 0.01	0.141 ± 0.02	0.004
Half-life (h), mean ± SD	8.7 ± 0.6	6.5 ± 0.9	0.03
Volume of distribution (L), mean ± SD	57.6 ± 2.6	71.8 ± 11.3	0.23

## Discussion

This study is the first to our knowledge to evaluate alterations in vancomycin pharmacokinetics due to enhanced renal clearance in patents with hemorrhagic stroke. All patients with hemorrhagic stroke included in the study experienced enhanced renal clearance, and the majority experienced ARC while concomitantly receiving vancomycin. Additionally, these patients exhibited pharmacokinetic alterations favoring an increased elimination of vancomycin when compared with predicted pharmacokinetic parameters based on population data. These findings advance the theory that patients with hemorrhagic stroke may require empiric renally eliminated medication dosing regimen modifications due to experiencing enhanced renal clearance.

Enhanced renal clearance and ARC are thought to be due to a hyperdynamic state experienced by some subsets of critically ill patients, resulting from systemic inflammation, administration of intravenous fluids and vasoactive medications, and increased organ blood flow [14]. There have been a few previous studies documenting enhanced and augmented renal clearance in patients with hemorrhagic stroke. In a study evaluating 20 patients with subarachnoid hemorrhage, May and colleagues found that the mean measured creatinine clearance among the patients included was 325.9 ± 135.2 mL/min/1.73 m^2^. This was significantly higher than the Cockcroft and Gault estimated creatinine clearance of 144.9 ± 42.8 mL/min/1.73 m^2^ (*p* < 0.001) [20]. One limitation from this study was that the creatinine clearance was calculated via one 24-h urine collection. Our research team expanded on these findings by measuring creatinine clearance daily in 80 patients with hemorrhagic stroke (50 patients with aSAH and 30 patients with ICH) via an 8-h urine collection [10]. We found that patients with aSAH or ICH had mean measured creatinine clearances significantly higher over the study period than the creatinine clearance estimated via Cockcroft and Gault. Ninety-four percent of patients with aSAH and 50% of patients with ICH experienced ARC on at least one study day.

Our research team has also previously demonstrated that patients with hemorrhagic stroke exhibited pharmacokinetic alterations favoring an increased elimination of vancomycin when compared with predicted pharmacokinetic parameters based on population data [21]. In a study including 146 patients with hemorrhagic stroke (80 with aSAH and 66 with ICH), the mean calculated elimination rate constant was higher than the predicted value (0.122 ± 0.04 vs. 0.086 ± 0.03 h^−1^; *p* < 0.001) and the mean calculated half-life was lower than predicted (6.4 ± 2.2 vs. 8.9 ± 3.1 h; *p* < 0.001). These alterations in pharmacokinetic parameters resulted in vancomycin serum trough concentrations lower than predicted (10.3 ± 4.3 vs. 18.3 ± 8.6 ug/mL; *p* < 0.001). It has also previously been proposed that renally eliminated medications may be eliminated more quickly than expected in patients who experience augmented renal clearance, resulting in subtherapeutic medication concentrations [22–25]. However, this is the first study demonstrating a direct association between elevations in creatinine clearance in patients with hemorrhagic stroke and increased elimination of vancomycin. These data reflect an important finding, as subtherapeutic vancomycin concentrations may lead to treatment failure and other serious patient complications. It is reasonable to postulate that these findings also apply to other renally eliminated medications. The concern of administering inadequate renally eliminated medication regimens may be of particular concern in patients with ICH, as these patients generally empirically receive dose reductions given their older age and higher likelihood of co-morbidities associated with renal dysfunction, such as hypertension and diabetes [26]. The findings from this study suggest that it may be acceptable to empirically dose patients suspected of experiencing ARC at higher doses than calculated with traditional population-based pharmacokinetic equations. Furthermore, these findings also highlight the need for additional studies to investigate new vancomycin dosing algorithms and empiric pharmacokinetic equations for patients with aSAH or ICH.

This study has several limitations worthy of discussion. There were a limited number of patients who had daily measured creatinine clearances and vancomycin serum peak and trough concentrations. Nevertheless, this is the first study to evaluate the impact of enhanced renal clearance on renally eliminated medication pharmacokinetic parameters in patients with hemorrhagic stroke. Additionally, limited information was available on follow-up vancomycin dosing regimens and subsequent serum concentrations, and the impact on infectious outcomes as vancomycin was frequently discontinued in an effort to narrow antibiotic therapy. However, this study illustrates that enhanced renal clearance in patients with hemorrhagic stroke results in vancomycin pharmacokinetic alterations, which also raises questions regarding the proper dosing of other renally eliminated medications that do not undergo routine therapeutic drug monitoring. This is an important area for future study.

## Conclusions

This is the first study to evaluate the impact of enhanced renal clearance on alterations in vancomycin pharmacokinetic parameters in patients with hemorrhagic stroke. Patients with hemorrhagic stroke exhibited a mean measured creatinine clearance greater than that estimated and pharmacokinetic parameter alterations favoring a faster elimination of vancomycin, resulting in subtherapeutic concentrations. These findings indicate that it is reasonable to measure CrCl via an 8-h urine collection in this patient population in order to properly dose adjust renally eliminated medications while also highlighting the need for future studies to determine optimal renally eliminated medication dosing strategies.

## Data Availability

Please contact author for data requests

## Abbreviations

ARC: Augmented renal clearance
aSAH: Aneurysmal subarachnoid hemorrhage
CrCl: Creatinine clearance
GCS: Glasgow coma score
ICH: Intracerebral hemorrhage
ICU: Intensive care unit
LOS: Length of stay
PK: Pharmacokinetic
SOFA: Sequential Organ Failure Assessment
